# Acoustic fine structure may encode biologically relevant information for zebra finches

**DOI:** 10.1038/s41598-018-24307-0

**Published:** 2018-04-18

**Authors:** Nora H. Prior, Edward Smith, Shelby Lawson, Gregory F. Ball, Robert J. Dooling

**Affiliations:** Department of Psychology, University of Maryland, College Park, USA

## Abstract

The ability to discriminate changes in the fine structure of complex sounds is well developed in birds. However, the precise limit of this discrimination ability and how it is used in the context of natural communication remains unclear. Here we describe natural variability in acoustic fine structure of male and female zebra finch calls. Results from psychoacoustic experiments demonstrate that zebra finches are able to discriminate extremely small differences in fine structure, which are on the order of the variation in acoustic fine structure that is present in their vocal signals. Results from signal analysis methods also suggest that acoustic fine structure may carry information that distinguishes between biologically relevant categories including sex, call type and individual identity. Combined, our results are consistent with the hypothesis that zebra finches can encode biologically relevant information within the fine structure of their calls. This study provides a foundation for our understanding of how acoustic fine structure may be involved in animal communication.

## Introduction

One of the great challenges facing ethology since its inception has been to understand the sensory and perceptual world an organism lives in. Von Uexkull famously postulated the concept of the “umwelt” which refers to aspects of the environment and surroundings that an animal attends to in order to survive and reproduce^1^. All animals filter the complex stimuli they encounter in the natural world through their species-specific sensory and perceptual capabilities. As we are reminded from the relatively recent research on vision, the perceptual abilities of non-human animals often are drastically different than our own. Whereas humans only have 3 cone-opsin proteins (the main determinant for cone cell sensitivity) in the eye which underlie color vision, many avian species have 4 (e.g.^2^) and fish can have 5–10 (*reviewed in*^3^). This highlights the fact that even for common species, we are continually expanding our basic understanding of a species’ “umwelt” and growing in our appreciation of how species differ in their perceptual abilities.

The fact that humans share a similar hearing range with birds (i.e. have overlapping audiograms) does not necessarily mean that bird vocalizations otherwise sound the same to us as they do to birds^4^. It is well known that perception can be selective. This notion is reflected by the concept of the sign stimulus, namely that only certain aspects of a complex stimulus may be perceptually relevant and capable of eliciting a behavior. In animal communication, playback studies and other behavioral investigations have clearly demonstrated that birdsong elicits strong behavioral responses across social contexts. Song has many acoustic features that result in perceptual attributes that are salient to humans (e.g. loudness, pitch, and tempo); however, it is an unresolved question whether the specific acoustic cues that birds are hearing are the same or different than what humans hear. In fact, it has been suggested that human percepts of pitch and even timbre may not be useful in understanding how birds perceive acoustic signals^5^.

Psychoacoustic experiments have shown that, compared to humans, birds are remarkably sensitive to variability in the fine structure (i.e. rapid modulations of the time-waveform or periods) of their vocalizations compared to humans (*reviewed in*^4,6^); however, there have been relatively few investigations into the more difficult questions, such as describing natural variation in the fine structure of vocal signals and/or identifying the potential functions of fine structure in vocal communication. Amongst birds, zebra finches appear to be especially sensitive to these subtle changes in fine structure^6,7^. Zebra finches can discriminate changes in fine structure of a harmonic complex occurring within an interval of 1 ms^6,7^, and this ability appears to extend to the fine structure of call-like stimuli synthesized from a single period from a natural female distance call^8^. More specifically, zebra finches are able to discriminate between two complex harmonic stimuli in which as few as ~5% of periods are reversed (7 periods)^6,8^. Fine structure as we define it is also reflected in the relative amplitude of individual harmonics, broadly relatable to timbre, and zebra finches are also sensitive to manipulations of the relative amplitude of harmonics^9–12^, especially of lower harmonics^10,12^. The limits of this discriminatory ability remain unclear, especially with regards to the acoustic variation in natural vocal signals, as well as how such perceptual abilities contribute to acoustic communication in this species.

There are many challenges to studying fine structure^13^, and the majority of research on fine structure has been conducted in humans. We know from research in humans that it is hard to separate the faster moving changes in amplitude associated with fine structure from envelope cues, and that it is unclear how fine structure is encoded and processed centrally. Despite these challenges in studying fine structure, evidence has accumulated to suggest that it is an important component of communication, at least for human speech^13,14^. Note that in this paper we will use temporal fine structure with respect to human research and acoustic fine structure with respect to non-human animal research due to subtle differences in the methodologies and frameworks used between the human and animal research.

In human speech, temporal fine structure may help a listener attend to, recognize and comprehend speech from a particular speaker within a group of speakers; this effect is especially apparent when a listener is asked to attend to predictable speech or familiar talkers against a noisy background^13,15,16^. In non-human animals, acoustic fine structure may function similarly. It is perhaps not surprising that zebra finches are particularly sensitive to changes in fine structure because their species-specific vocalizations are harmonic stacks which are inherently rich in fine structure. Related to this, manipulating the fine structure of zebra finch calls by changing the relative amplitude of harmonics, results in subsequent changes in zebra finch behavioral responses during naturalistic playback experiments^9^. If acoustic fine structure is functionally relevant in zebra finch communication, then it raises other interesting questions such as whether sensitivity to fine structure has coevolved with the ability to produce an equivalent degree of richness in fine structure in natural vocalizations^4^.

The goal of our current study is to examine, as much as possible, acoustic fine structure independent of other acoustic characteristics within the natural vocal signals of zebra finches. Our focus here is to examine acoustic fine structure in the temporal domain, rather than its spectral correlate, allowing us to identify and manipulate the smallest ‘units’ of acoustic fine structure, periods, as we have done previously^8^. Together these experiments provide a foundation for our understanding of how acoustic fine structure might function within natural communication systems of non-human animals.

## Results

### Natural variation in acoustic fine structure (periods) within and between calls

**Figure 1:**
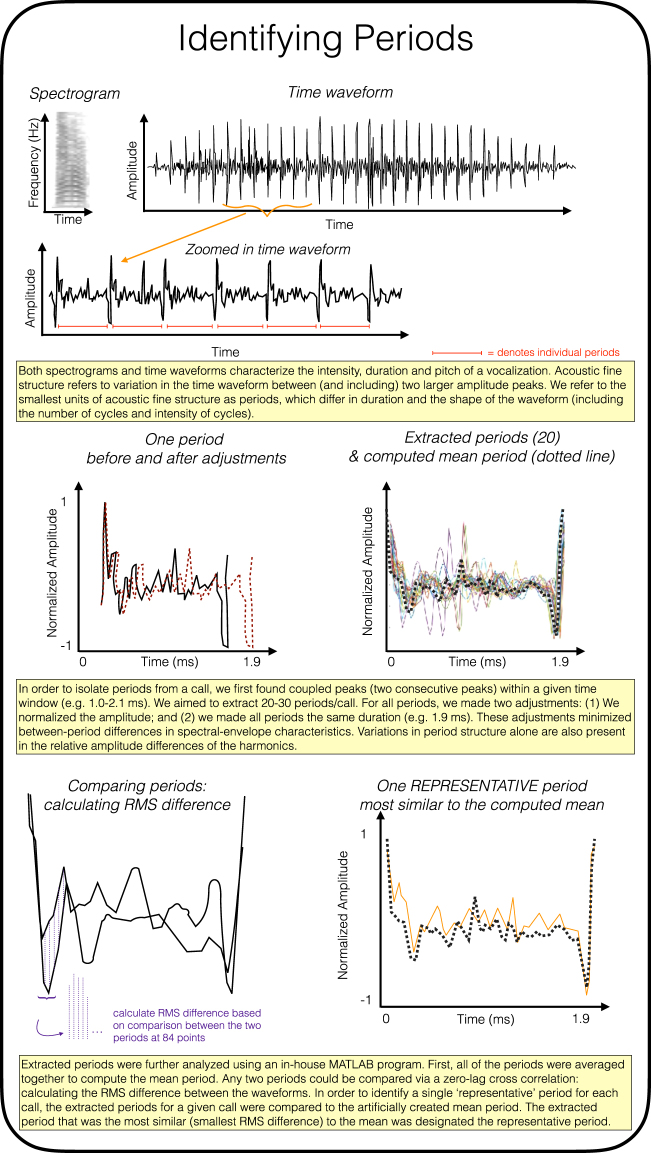
Schematic representation of the period isolation and extraction methods.

Here we defined individual periods as the time-waveform between two consecutive larger amplitude peaks (Fig. 1). For a subset of 16 calls (consisting of male short calls, female short calls, male distance calls and female distance calls), an average of 30 periods/call (min = 16, max = 38) were extracted based on our criteria (two consecutive peaks within 1.0–2.1 ms). These extracted periods were adjusted in duration (1.9 ms) and the amplitude was normalized. These two adjustments minimized spectral and envelope differences.

**Figure 2:**
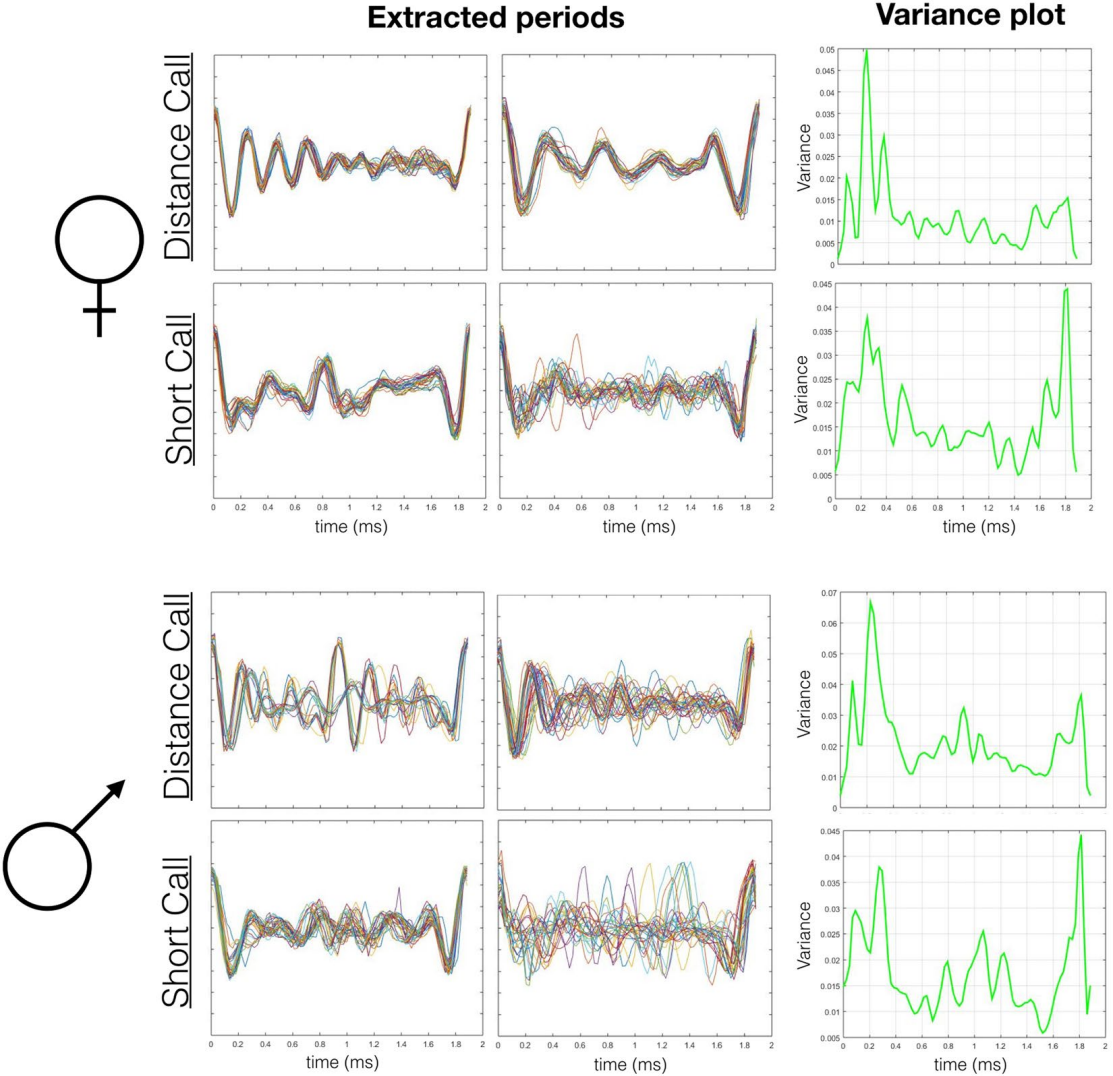
In the first two columns, each panel depicts 10 overlaid periods extracted from a single call. Each row is a different call type within females and males. Additionally, the third column plots variance in period structure across time. Each variance plot is based on up to 20 periods extracted from 9 different calls from 3 different individuals. Note that the y axes for the variance plots differ. There was the most variance for male distance calls (y-max = 0.07).

Using root-mean-square (RMS) differences as an estimate of the similarity in the structure between two periods, we compared intra- and inter-call variation. In general, period structure varied more between calls than within calls. However, there was substantial variation in period structure within a call (Fig. 2; Supplementary Fig. 1).

In addition to using the RMS difference, we also plotted the variance of period structure calculated from 9 calls/call type from several individuals (Fig. 2). Across call types, there was greater variance at ~0.3 ms at the beginning or end of a period. However, this is not surprising since the largest amplitude portions of a period are at the beginning and end. Additionally, there is greater variance for periods from distance calls compared to short calls.

Our subsequent experiments are a combination of psychoacoustic methods, in order to test the extent to which fine structure variation in vocalizations can be detected and discriminated, as well as signal analysis methods, to investigate whether there may be biologically relevant information encoded within acoustic fine structure. For these subsequent experiments, we focused on a single representative period. The representative period was the naturally occurring period that most closely resembled the structure of a computed mean period for any given call (Fig. 2).

### Discrimination of within-call variation in period structure

In order to determine how the zebra finch perceives natural variation in periods, we first aimed to identify whether the variation in period structure within a single vocalization was discriminable (Experiment 1). Four zebra finches (two males and two females) were tested using our psychoacoustic paradigm (*see methods*) on four different stimulus sets generated by concatenating a single period from each of four natural calls to create 200 ms call-like stimuli. For all birds, performance was greater than 50% correct within 1–3 steps from the representative period.

**Figure 3:**
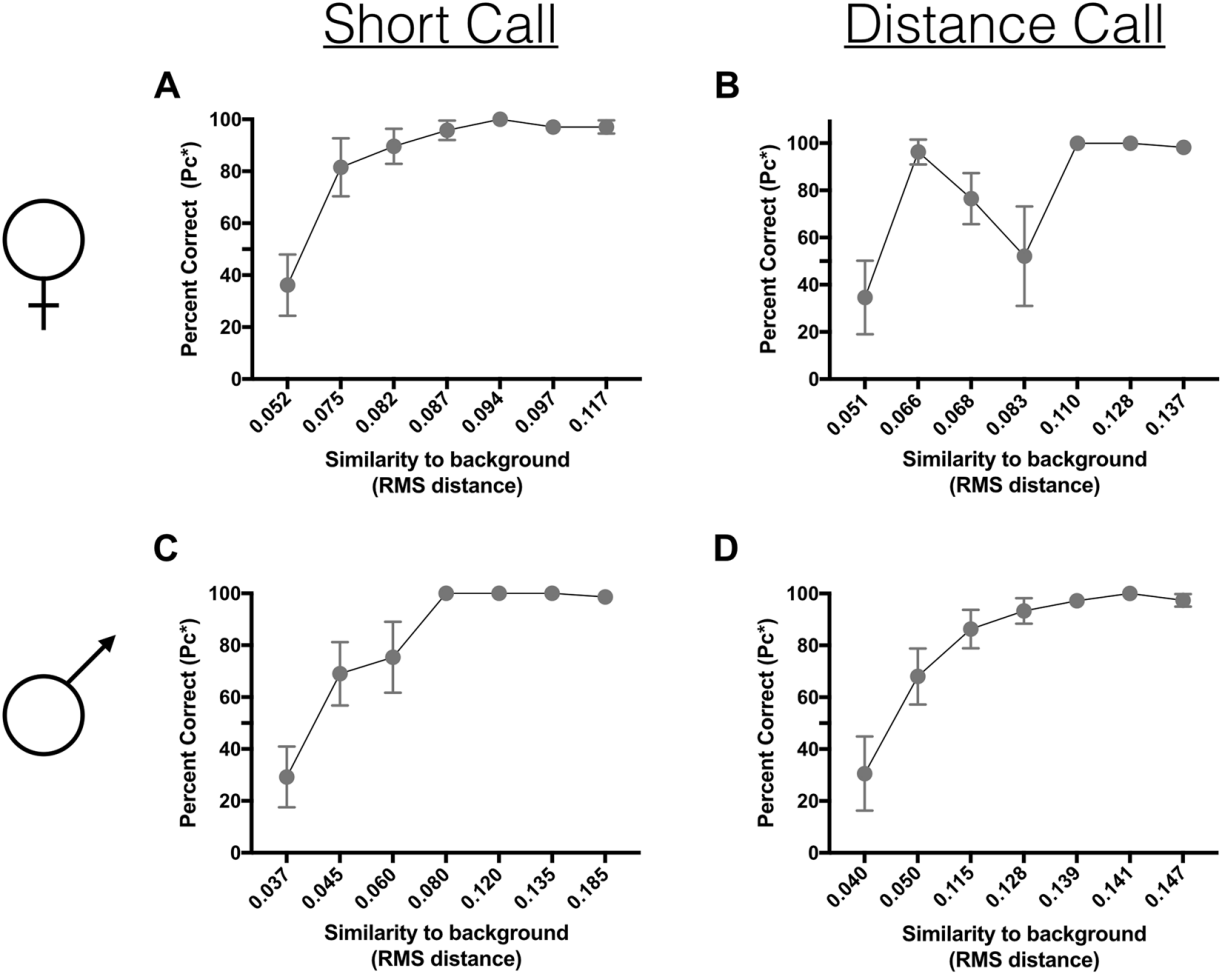
Zebra finches were able to discriminate some of the smallest changes in the structure of periods naturally produced within a call. This is true for periods taken from: (**A**) a female short call; (**B**) a female distance call; (**C**) a male short call; and (**D**) a male distance call.

Importantly, the birds showed similar thresholds for discriminating structural variation across periods for four stimulus sets from different vocalizations taken from different classes of call types (male distance call, male short call, female distance call, and female short call). Results show that zebra finches were able to discriminate some of the smallest changes in period structure that we show are present in their calls (RMS value ≤ 0.05 in some cases) (Fig. 3A–D).

### Discrimination of variation in random arrangement of multiple periods within a call

**Figure 4:**
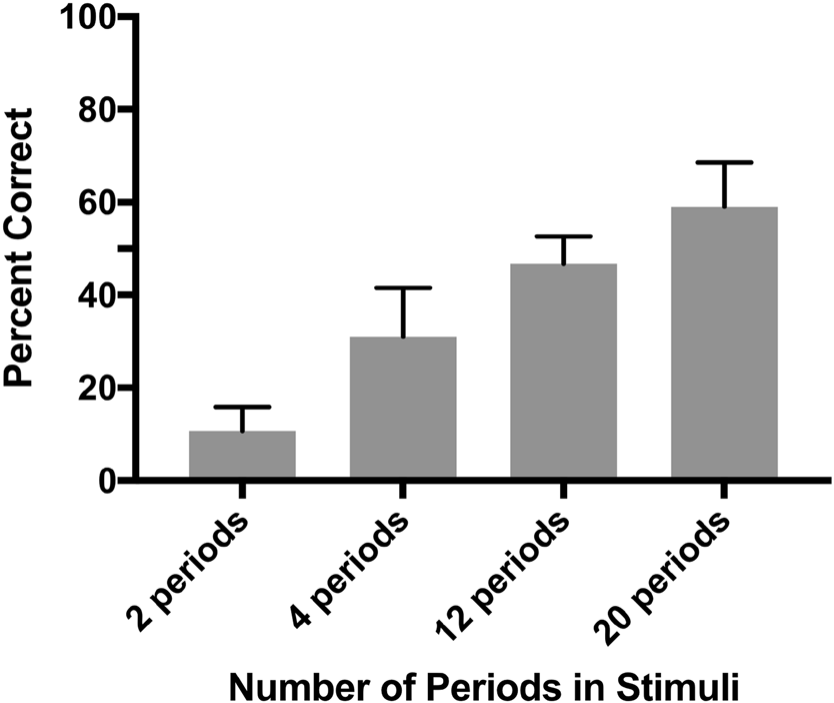
For stimuli composed of 20 periods, zebra finches were able to discriminate between different random arrangements of periods.

Next, we tested whether zebra finches could discriminate changes in the arrangement of periods within a call, and whether this discriminatory ability differed depending on the number of periods (Experiment 2). Here call-like stimuli were generated from either 2, 4, 12 or 20 different periods extracted from a single female short call. These periods were concatenated in each stimulus in random order. The zebra finch’s ability to discriminate different arrangements of periods improved as the number of periods increased (Fig. 4). When 2, 4 or 12 periods were used, performance fell below 50% correct, but when the stimuli were made up of 20 periods, zebra finches could discriminate among stimuli with a different random order of periods well above 50% correct. This finding, together with the finding that these birds can discriminate amongst some of the smallest naturally occurring differences in period structure (Experiment 1), strongly suggests that variation in the acoustic fine structure of these harmonic vocalizations might carry biologically relevant information that could be salient to both male and female zebra finches.

### Discriminable variation in period structure is also represented in the spectral domain of signals

**Figure 5:**
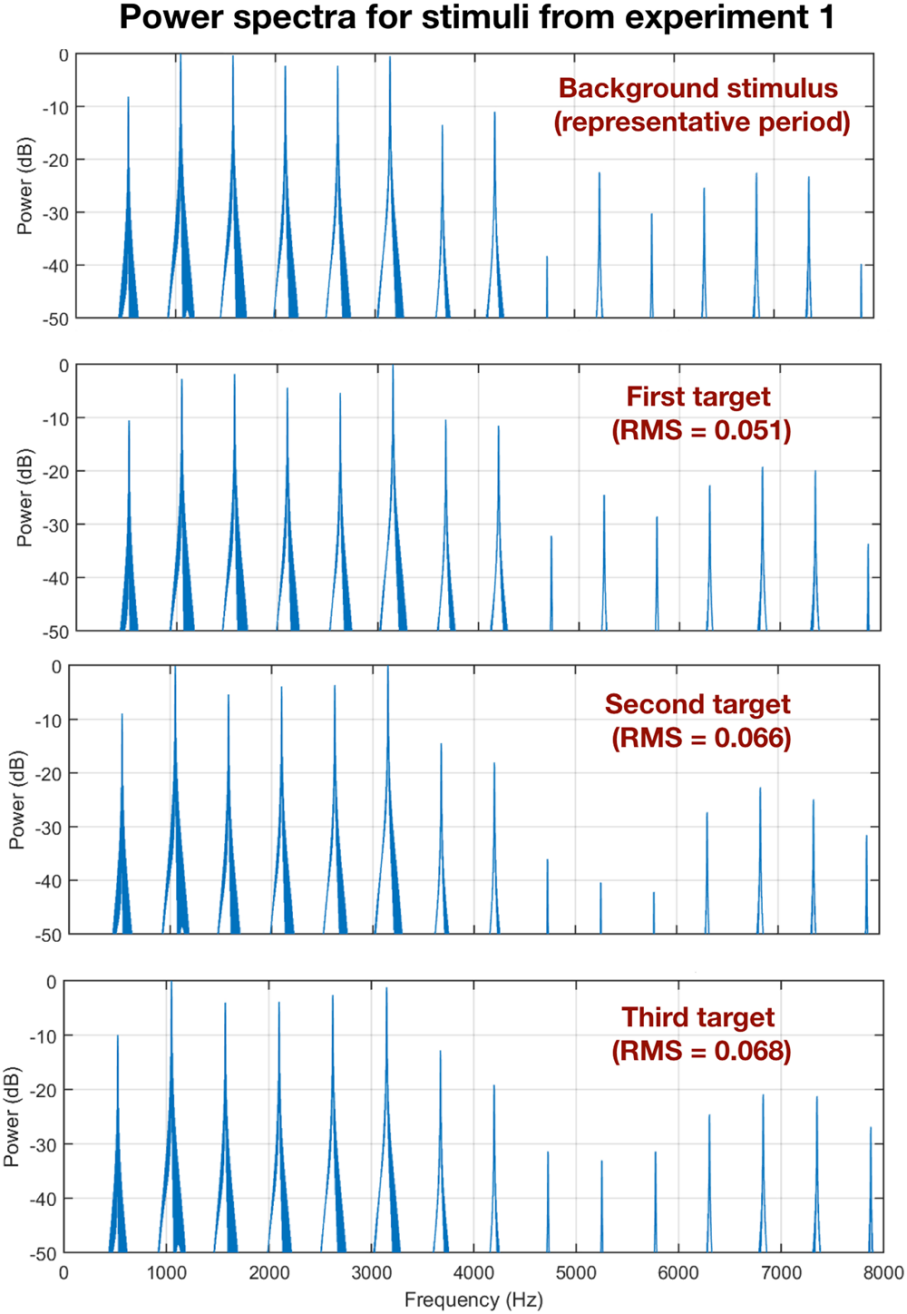
Power spectra for three call-like stimuli used in Experiment 1. Each stimulus was composed of a single period from a female distance call.

Spectral analyses of the stimuli that we generated for Experiment 1 and 2 (above) show that the period variation in acoustic fine structure of natural vocalizations is represented in the spectral domain as differences in the relative amplitude of harmonics (Fig. 5). Consistent with this, there is evidence that temporal fine structure is more important than envelope cues for determining pitch perception in humans^17^. Furthermore, casual human listeners report subtle differences in pitch, roughness, and timbre among the stimuli used in Experiments 1 and 2.

**Figure 6:**
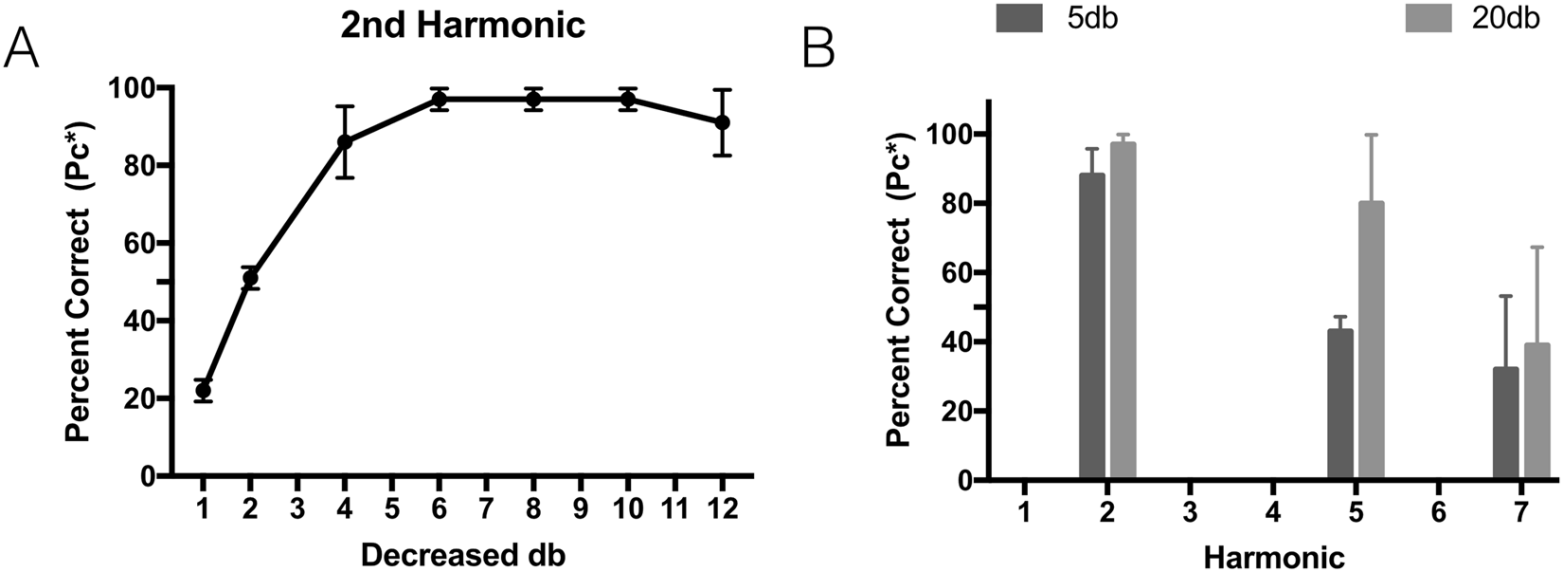
Using an artificial harmonic complex, we determined zebra finches could discriminate small changes in the relative amplitude of harmonics: (**A**) as small as ~3 db at the 2^nd^ harmonic, and (**B**) and between 5–20 db at 5^th^ and greater than 20 db at the 7^th^ harmonic.

As a test of the degree to which such harmonic amplitude differences are discriminable, we conducted two more psychoacoustic experiments (Experiments 3 and 4). For both of these experiments, two zebra finches (1 male and 1 female) were tested on their ability to discriminate differences in the relative amplitude of harmonics in a synthetic harmonic complex (with a fundamental frequency of 570 hz). First, zebra finches were tested on 7 levels of decreasing intensity in the 2^nd^ harmonic. The threshold for detecting a decrease in the amplitude of the 2^nd^ harmonic was about 2 dB (Fig. 6A). Second, a separate experiment looked at the sensitivity across harmonics by reducing the amplitude of the 2^nd^, 5^th^, and 7^th^ harmonic by either 5 or 20 dB (Fig. 6B). Performance was worse at these higher frequency harmonics and fell below 50% for the 7^th^ harmonic. These results are consistent with earlier work showing zebra finches can discriminate extremely small amplitude differences in these specific harmonics^10,12^.

### Evidence that sex and call type may be encoded within the acoustic fine structure

The results of Experiments 1 and 2 demonstrate that zebra finches can discriminate extremely subtle, moment-to-moment variation in the fine structure of naturally produced calls, raising the question of whether biologically relevant information is reflected in fine structure. In order to tackle this question, we used two complementary analyses. These analyses were conducted on calls from 18 zebra finches (9 males and 9 females). For each individual, a total of 20 calls: 10 short calls (stack or tet, which represent a continuous category) and 10 distance calls were used^18,19^. For every call, we identified the single representative period closest to the computed mean to use for subsequent analyses. Thus, the analyses are only on a representative portion of the acoustic fine structure.

First, we used half of our periods to train a support vector machine (SVM), and then tested this classification ability using the second half of our calls. This was done iteratively, 1000 times, in a Monte-Carlo scheme in order to estimate the effectiveness of our SVM for classifying periods by sex (male vs female) and call type (distance call vs short call) (*see methods)*. In most instances, periods were correctly classified based on their acoustic fine structure to their biological category. For sex, 70.0% of periods were correctly assigned as male or female. For call type, 77.7% of periods were correctly classified as distance or short calls.

Secondly, we also used RMS difference measures as an estimate of the structural similarity between two periods. A series of pairwise comparisons was made to determine whether periods within a biologically relevant category were more similar than periods between categories (sex, call type). Periods were more structurally similar (smaller RMS value) within females than between females and males (p < 0.0001). Interestingly, period structure was more variable (higher RMS difference) within males than within females. For call type, periods were more structurally similar within a call type: distance calls were more similar to distance calls and short calls were more similar to short calls (p < 0.0001).

The fundamental frequency, estimated from the duration of the representative period, did not differ between males and females or by Call Type (Sex χ^2^(1) = 2.49, P = 0.114; Call type χ^2^(1) = 1.15, P = 0.284; Sex × Call type χ^2^(1) = 0.64, P = 0.421). However, when we looked for an effect of sex on fundamental frequency separately for short calls and distance calls, we found that the fundamental frequency was higher in males than females for distance calls alone (χ^2^(1) = 5.21, P = 0.022). There was no difference based on sex for short calls (χ^2^(1) 0.71, P = 0.400). That male distance calls have a relatively high fundamental frequency is consistent with previous research^19^.

### Evidence that individual identity may be encoded in acoustic fine structure

We used the same group of calls (20 calls each from 18 individuals, 9 males and 9 females) in order to ask whether the acoustic fine structure reflected individual identity. Here we did not have the appropriate sample size to build a SVM for 18 categories. Thus, we only used the pairwise comparisons of RMS difference values between any two periods as described for sex and call type. Due to the number of comparisons for this analysis, we used an alpha of 0.01 and also more fully estimated the baseline noise floor using a Monte-Carlo simulation (*see methods*). More specifically, for our dataset, we estimated that 17% of comparisons would be significant by chance (*see methods*). Using this pairwise comparison method, we found that for 63% of the comparisons, period structure was more similar within individuals than between individuals (RMS difference).

## Discussion

Here we describe natural variability in the acoustic fine structure within and between calls; we show that such natural variability is discriminable; and we provide evidence that fine structure may carry biologically relevant information. Combined, our results are consistent with the hypothesis that the zebra finch’s perceptual sensitivity to acoustic fine structure is matched to the ability of the syrinx to produce variability in fine structure, as represented by periods in the time-waveform. This match of the acoustic fine structure produced to the auditory discriminatory ability of zebra finches suggests that the acoustic production and perception systems may have co-evolved based on the communicative advantage that fine structure serves. Our study outlines a foundation for this hypothesis by showing that acoustic fine structure in these vocalizations may carry biologically relevant information about the sex, call type and identity of the caller, and that this encoded information is likely salient for zebra finches. Similar approaches could be used to determine whether other behaviorally relevant information is encoded within acoustic fine structure.

It should not be surprising that bird calls, such as the zebra finch short call, have rich communicative potential. Peter Marler emphasized the point that bird calls carry significant behavioral relevance beyond our current knowledge^20,21^. While calls have historically been considered to be inflexible and less socially enriched than songs^20^, there is growing evidence from both behavioral and neurobiological studies that suggest bird calls have rich communicative potential^19,22,23^. Continuing investigations of the relevant information from a vocal signal may reveal other acoustic features that lie below human sensitivities that are nevertheless used by birds to communicate important information.

Marler emphasized that it is critical for researchers to re-examine the functional role and neurobiological basis of bird calls^20^. Our research suggests that acoustic fine structure may provide an exceedingly rich, and heretofore unexplored parameter within the vocal communication channel. Traditionally, other bioacoustics approaches have investigated complex acoustic signals using computational techniques such as potential for individual coding (PIC) and principal component analysis (PCA). For PIC analyses, the coefficients of variation for specific acoustic features are compared within and between biologically relevant categories (typically individuals), in order to determine the likelihod that a particular acoustic feature differs across categories (e.g.^24–27^). In contrast, PCAs can be conducted based on a suite of acoustic features  (e.g.^19,28,29^). However, this type of dimensionality reduction produces scalar components that are not easily related back to specific acoustic characteristics of a signal or to perceptual abilities. To complement these computational methods, animal discrimination abilities have been assessed via experimental methods (either via naturalistic playback and/or various operant methods) that use manipulated acoustic stimuli from between vs within categories. By making gross perturbations to signals (such as removing harmonics or adjusting the temporal envelope), researchers have aimed to identify the acoustic characteristics of a signal that carry biologically relevant information (e.g.^30–33^). Combined, these methods are useful for relating general acoustic features to behavioral contexts and/or neurobiological representations (e.g.^19,22,34,35^). However, our results suggest that additional methods that maintain more of the richness of acoustic information present in signals but rely less on dimensionality reduction may be useful in order to more fully understand the production-perception relationships at a mechanistic level and to determine how complex information is encoded within a signal.

Zebra finches represent an ideal study system: not only are they particularly sensitive to acoustic fine structure, as illustrated by their ability to discriminate variation in such stimuli, but they also produce calls that are strongly harmonic and therefore rich in fine structure. The variation that exists in fine structure likely results from subtle changes in the avian vocal tract including syrinx as well as upper vocal tract filter properties (including tracheal properties, esophageal-oropharyngeal cavity and beak movements)^36–38^. Experimental manipulations of the syrinx and vocal tract properties can be used to identify what aspects of the production mechanisms relate to the acoustic fine structure present in vocal signals. Mencio *et al*. (2016) reduced neuromuscular transmission via local pharmacological manipulation of the syrinx, which caused a reduction in frequency modulation rates and eliminated some high-frequency sounds without changing the fundamental frequency of syllables^39^. However, it is not clear how such high frequency modulations are relatable to acoustic fine structure. Furthermore, twitch kinematics of the syrinx muscles of the zebra finch are some of the fastest “superfast” muscles known in any vertebrate species^40,41^. Taken together, these recent studies on the zebra finch syrinx, along with our current findings, raise interesting questions about how these perception-production abilities co-evolved and what selection pressures supported the evolution of such a rich communication channel.

As stated earlier, here we only tried to identify evidence of whether acoustic fine structure could distinguish between relatively general biological categories. There is a significant amount of behavioral and neurobiological research that has demonstrated that zebra finches are able to discriminate the calls of males and females^18,22,29,42^. Additionally, zebra finches have 10 call types in their call repertoire, and there is strong physiological evidence that perceptual categories exist for many of those call types^22^. Furthermore, there is also independent evidence that zebra finch calls carry individual identity, which is at least accessible to an individual’s pair-bonded mate (e.g.^29,43–45^). This evidence exists for both the distance call, which is largely used when birds are not in visual contact, but also for short calls, which are used when birds are within visual contact^29,46^. However, it is not known whether all zebra finch short calls carry information on individual identity. A recent study found evidence that zebra finches can identify individuals using the more harmonic stack call, but not other short calls^29^. Interestingly, the short calls we used here were more stack-like (longer, more harmonic, and less frequency modulated) than tet-like, thus it is unclear to what extent our findings extend across all short calls.

To be clear, we are not claiming that changing a single 2 ms period within a complex vocalization is perceptible to zebra finches, rather we are suggesting that variation across small subsets of periods may be discriminable. The fact that we see acoustic evidence of biologically relevant categories carried within a single period (<2 ms) of a call is remarkable. This raises the question of whether acoustic fine structure can be used to identify other types of information that may exist within a call.

Beyond carrying information that enables the bird to distinguish amongst general biological categories such as those investigated here, zebra finches could communicate many additional types of information. Zebra finch pairs remain together throughout their lives, actively maintaining their pair bond regardless of breeding condition^18,47^. Additionally, they breed opportunistically due to the unpredictable climate in central Australia, carefully coordinating the timing of breeding bouts^18^. Thus, based on zebra finch’s ecology, it is not unreasonable to expect that information about the internal state of an individual’s partner would be important. Indeed, there is evidence that zebra finch calls exhibit such flexibility and that the characteristics of the spectral envelope reflect motivational state^34,48–50^. Importantly, within this framework, calls act as honest signals^51^, meaning that if the vocal signal and production mechanisms are flexible enough the signal could be influenced by subtle aspects of an individual’s physiological state. Thus, behaviorally relevant information may be present and salient within calls without the vocalizer intentionally encoding such information. At least for the zebra finch, our current results support the hypothesis that acoustic fine structure of vocal signals is variable and thus may be responsive to internal state and that the zebra finch auditory system is equal to the task of decoding such information. Future research will determine to what extent fine structure reflects other aspects of internal state (e.g. stress levels, motivational state, breeding state).

The problem of identifying specific acoustic features responsible for communicating complex information is a general question in neuroethology, as well as in speech communication. For instance, while envelope cues seem to carry the majority of speech information, temporal fine structure is extremely important for communication. More specifically, age-related hearing deficits that make it difficult to carry on conversations in loud, crowded spaces may be associated with deficits in temporal fine structure processing^13,14^. For non-human animals, the production and perception of acoustic fine structure is largely unexplored (with some exceptions^52^). Our current study highlights that there remain many outstanding questions about the role of fine structure in communication across species.

## Methods

### Ethics Statement

Animal husbandry and experimental procedures used here were approved by the University of Maryland Animal Care and Use Committee (protocol number: 951721-10 and 689824-8). Additionally, these procedures also followed the Animal Behavior Society (ABS) and Acoustical Society of America (ASA) guidelines for the use of animals in research.

### Subjects

For Experiments 1–4, zebra finches were housed individually, in visual and auditory contact with each other and other birds throughout the experiment. These birds were mildly food deprived (~90–95% of free feeding weight) and kept on an 8L:16D light cycle throughout the duration of the experiments. For Experiments 1 and 2 we tested 2 males and 2 females on our stimulus sets. For Experiments 3 and 4, we tested 1 male and 1 female, which also had been used for Experiments 1 and 2. Additionally, we recorded calls from 2 other zebra finches (1 male and 1 female) to generate the acoustic stimuli used for the psychoacoustic experiments (1–4).

For Experiment 5, the zebra finches (9 males and 9 females) that were recorded were housed with their pair-bonded mate, in a separate colony room. Here zebra finches were housed with *ad libitum* seed, water and grit on a 12L:12D light cycle.

### Recordings

Acoustic fine structure can be directly affected by the environmental conditions that were present during recording. Our recordings were conducted under the same conditions. All recordings were conducted in an acoustically-treated room using tie-clip microphones (AKG C417) and a zoom F8 multitrack field recorder (44,100 Hz sampling rate). Recordings were made in small cages where the birds were close to microphones (5–10 inches). Calls were identified and extracted manually in Adobe Audition (ver: 9.2.0.191). Finally, we checked that no vocalizations were clipped in our recordings.

#### Period Extraction

For signal analysis and the preparation of stimuli for psychoacoustic tests, single periods were identified and extracted from natural zebra finch calls using custom MATLAB software (See Fig. 1 for summary of methods). To do this, the program first identified the 40 largest amplitude peaks within each time waveform (each call), and then located coupled peaks, i.e., two peaks separated by a specific temporal window (psychoacoustic test 1.0–2.1 milliseconds; signal analysis 1.4–2.0 milliseconds). Note that for the signal analysis we used a slightly more conservative temporal window, to minimize artifacts due to artificial stretching of periods. A period was defined as the time waveform between (and including) the two coupled peaks. Approximately 20 periods were selected from each call.

To minimize differences in amplitude and fundamental frequency (as well as harmonics of the fundamental frequency), two adjustments were made to each period. First, the peak-to-peak amplitude of each period was scaled to a value of one. Second, the duration of each period was stretched or compressed to 1.9 milliseconds (1.7 for the signal analysis). After these adjustments, periods retained the relatively small structural features between the two peaks. These smaller features varied in number, position, and relative amplitude. The questions we tested here focused on whether differences in these smaller features were discriminable.

#### Identifying the Representative Period

Extracted periods were then further analyzed to identify the representative period for each call. A mean period was first computed by averaging together all of the adjusted periods from the call. To identify the representative period for each call, the RMS difference from each adjusted period to the mean period was computed. The RMS difference between any two adjusted periods is the root mean squared value of the differences between corresponding digital samples. For the two adjusted periods R and S, each having N digital sample points (for periods adjusted to 1.9 milliseconds, N was 84), the squares of the time-aligned differences are averaged, and the RMS difference between R and S is the square root of that average.

1 $${\rm{RMS}}\,{\rm{difference}}\,{\rm{between}}\,{\rm{R}}\,{\rm{and}}\,{\rm{S}}=\sqrt{\mu (RI-S{I}^{2})}$$

The extracted period that was closest to the mean (i.e., had the smallest RMS difference to the mean) was taken to be the representative period. Using the same RMS difference metric, all the periods from a given call could be rank-ordered based on how close they were to the representative period.

#### Psychoacoustics Experiment

Two psychoacoustic experiments were conducted to determine the extent to which zebra finches can discriminate natural variation in period structure. For Experiment 1, a background call-like stimulus was generated from the representative period of a given call. The 7 targets were then call-like stimuli generated from different periods from the same call that were increasingly less similar to the background (increasing RMS difference). This experiment used four different sets of stimuli generated from periods extracted from different call types (female short call, female distance call, male short call, and male distance call). For Experiment 2 stimuli were generated from multiple periods from a single call. The background and the two target stimuli were generated from the same periods but differed in the random arrangement of the periods within the stimuli. The four stimulus sets differed in the number of periods used to generate the stimuli (2, 4, 12 or 20).

Two additional psychoacoustic experiments (Experiments 3 and 4) were conducted to determine whether the zebra finch’s sensitivity to period structure corresponded to a sensitivity to variation in the relative frequency of harmonics in an acoustic signal (as these temporal and spectral features are related). Here we constructed artificial harmonic complexes using an in-house MATLAB program, similar to what has been used previously with a fundamental frequency of 570 Hz and harmonics ranging from 1 kHz to 5 kHz^12^. The initial phase of all the harmonics was 0. The repeating background stimulus for Experiments 3 and 4 used a single harmonic complex with the relative amplitude of each harmonic equal. In Experiment 3, we decreased the 2^nd^ harmonic by 1 db, 2 db, 4 db, 6 db, 8 db, or 10 db to accurately assess the threshold for what harmonic amplitude changes zebra finches can discriminate. In Experiment 4, the 6 targets were harmonic complexes with either the 2^nd^, 5^th^ or 7^th^ harmonic decreased by 2 db or 5 db. The frequencies of these harmonics were chosen in order to be consistent with previous research examining the mistuning of harmonics^12^.

### Apparatus

This psychoacoustic paradigm has been described previously in detail^53,54^. Briefly, birds were tested in an apparatus consisting of an IAC-3 sound isolation chamber (Industrial Acoustics Company, Inc., Bronx, NY) housing a small cage (23 × 25 × 16 cm) and a response panel with two light-emitting diode (LED) microswitches. In-house MATLAB software controlled Tucker-Davis hardware (Tucker-Davis Technologies, Alachua, FL). Playback was conducted using a Crown D-75 amplifier (Crown International, Inc., Elkhart, IN) used to drive an Orb full range point source speaker (Mod1) which was placed 40 cm from the bird. All stimuli were resampled at 24,414 Hz and attenuated in MATLAB such that they played at ~65 dB in the apparatus. During testing, birds received food rewards from a hole on the ground of the cage, via an automated hopper.

### Procedure

This procedure we use is a discrimination task^53^. Here we trained birds to respond if they can “hear” a change in a given stimulus. Birds were trained to press one key (observation) during a continuous repeating background and press another key (target) when they hear any difference. This is very similar to a standard human hearing test when a subject is instructed to press a button when they hear a tone.

Zebra finches were trained to peck one LED, the observation key, while a sound (the background) was repeated continuously. After a random interval of time (2–7 s), a new sound (the target) alternated with the background sound. Birds were rewarded with a 2 s access to seed if they pecked a second LED, the report key, within 2 s of hearing the target sound (HIT). Failure to peck the report key within 2–3 s was scored as a MISS (note that 3 s was used for Experiments 1 and 2; and 2 s was used for Experiments 3 and 4). Thirty percent of all trials were sham trials where a target sound was not produced. Pecking the report key during a sham trial was scored as a FALSE ALARM resulting in a mild punishment of the lights being briefly extinguished in the test chamber. Following either a HIT, FALSE ALARM, MISS or CORRECT REJECTION, the software would initiate a new trial after the bird again repeatedly pecked the Observation key.

Birds typically ran in 100 trial sessions once or twice a day until their performance stabilized with a false alarm rate less than 25%. Typically, this occurred during the first 200 trials, but occasionally birds would be tested on additional sessions to meet this criterion. All further analyses were conducted on a measure of corrected percent correct (Pc*) for analyses to adjust for the effects of different false alarm rates according to the formula, where FA = false alarm, Pc = percent correct, and Pc* = corrected percent correct:

2 $${\text{Pc}}^{\ast }=({\rm{Pc}}-{\rm{FA}})/(1-{\rm{FA}})$$

We used Pc* = 50% as our definition of a threshold^55,56^. Importantly, this measure assessed each bird’s sensitivity while correcting for their individual response bias. For this study, the average false alarm rate across the 4 zebra finches and all the experiments was 11%.

#### Signal Analysis: Experiment 5

In order to determine to what extent acoustic fine structure carries biologically relevant information, we focused on the single representative period. We examined these periods in two different ways: firstly, we used all of the information in each period, based on the 84 sampling points, in order to train a support vector machine (SVM); secondly, we assessed how similar period structure was between every two periods using RMS difference measures. If period structure reflects biological categories, we would expect that period structure would be more similar within a category than between categories (smaller RMS difference = more similar). We used these approaches in order to determine whether calls could be classified to the sex of the vocalizer and/or the call type (data set = 10 short calls and 10 distance calls from 9 males and 9 females). We conducted Welch’s t-tests (alpha = 0.01), allowing for unbalanced comparisons, on the RMS values for each categorical comparison. For the comparison of sex there were N = 180 male and N = 180 female calls, and for the comparison of call type there were N = 180 short and N = 180 distance calls.

In order to determine whether the acoustic fine structure differed across individuals we were only able to use our second method of analysis. Calls were subdivided by individual identity (N = 20 calls/individual), by pooling call types. Again, RMS differences between any two periods was used to estimate whether the structural similarity of periods was more similar within or between individuals. Here 306 Welch’s t-tests were conducted for individual identity across the full call data set. Due to the number of statistical tests, we more completely estimated the baseline noise floor for this analysis. Here the full data set of calls (N = 360) was randomly distributed into arbitrary “categories”, and the same analysis was repeated. This procedure was then repeated 1000 times for N = 18 groups, 20 calls per group. For 17% of comparisons across the 1000 runs, the periods were more similar within a category than between categories. Thus, based on chance, we would expect 17% of these pairwise comparisons to be significant.

To determine whether the fundamental frequency of the representative periods contained relevant information, the fundamental frequency was estimated for the representative period based on its duration (prior to adjustment). These data were analyzed in R (v. 3.2.3, 2015, R Foundation for Statistical computing) using generalized linear-mixed models (LMMs; function lmer from the lme4 Package) with call type and sex as fixed factors and individual identity as a random factor. For each model, the distribution of residuals was checked prior to conducting the analysis and the data were transformed as necessary.

### Data availability statement

The datasets generated during and/or analyzed during the current study are available from the corresponding author upon reasonable request.

## Electronic supplementary material

Supplementary Figure
