# Supplementary material for: Acoustic fine structure may encode biologically relevant information for zebra finches
Source: Sci Rep. 2018 Apr 18;8:6212. doi: 10.1038/s41598-018-24307-0 (PMC5906677; doi:10.1038/s41598-018-24307-0)
Supplement: Supplementary file 1 — Supplementary Figure [file 41598_2018_24307_MOESM1_ESM.pdf]

Supplementary information:

Acoustic fine structure may encode biologically relevant information for zebra finches

Nora H. Prior[1,*], Edward Smith[1], Shelby Lawson[1], Gregory F. Ball[1], Robert J. Dooling[1]

[1] Department of Psychology, University of Maryland, College Park, USA

*Corresponding author
Biology-Psychology Bldg.
University of Maryland
4094 Campus Dr.
College Park, MD 20742
Email: nhprior@umd.edu

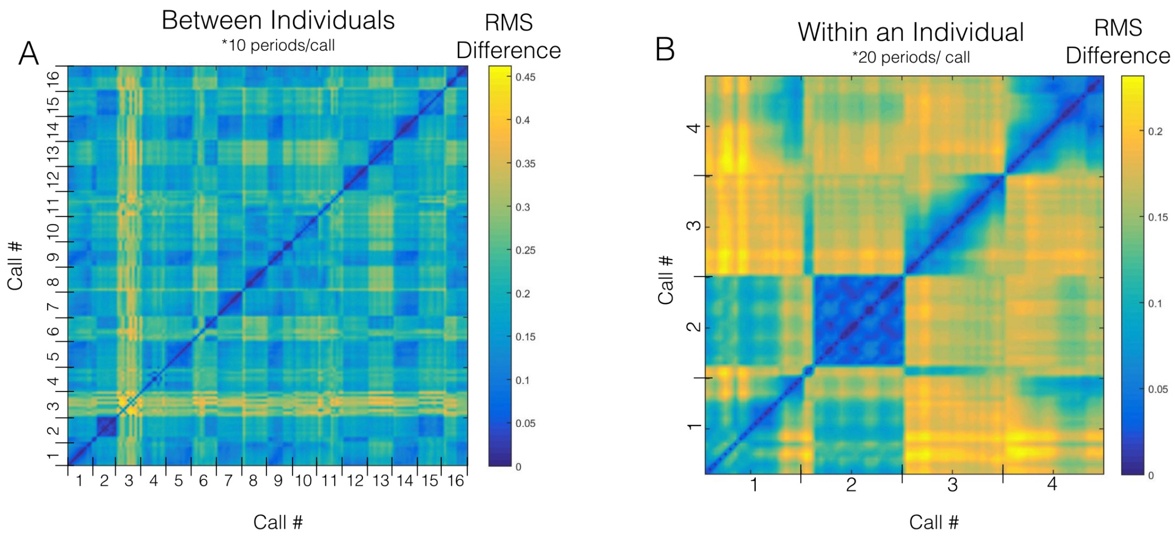

Supplemental Figure 1: Mosaic plots display variation in period structure, as estimated by RMS differences between any two periods, within and between individuals. Here multiple periods are displayed for each call. (A) Between individual variation is displayed (10 periods/call) for 16 different calls. (B) Within a single individual (female) period structure variability is displayed (20 periods/ call).
